# Co-creating and Evaluating a Web-app Mapping Real-World Health Care Services for Students: The servi-Share Protocol

**DOI:** 10.2196/resprot.6801

**Published:** 2017-02-16

**Authors:** Ilaria Montagni, Emmanuel Langlois, Jérôme Wittwer, Christophe Tzourio

**Affiliations:** ^1^ Univ. Bordeaux, Inserm Bordeaux Population Health Research Center, team HEALTHY, UMR 1219 F-33000 Bordeaux France; ^2^ Univ. Bordeaux, Centre Emile Durkheim Science politique et sociologie comparative, UMR 5116 F-33000 Bordeaux France; ^3^ Univ. Bordeaux, Inserm Bordeaux Population Health Research Center, team EMOS, UMR 1219 F-33000 Bordeaux France

**Keywords:** mapping of services, real-world health care services, Web-app, access to care, university students

## Abstract

**Background:**

University students aged 18-30 years are a population group reporting low access to health care services, with high rates of avoidance and delay of medical care. This group also reports not having appropriate information about available health care services. However, university students are at risk for several health problems, and regular medical consultations are recommended in this period of life. New digital devices are popular among the young, and Web-apps can be used to facilitate easy access to information regarding health care services. A small number of electronic health (eHealth) tools have been developed with the purpose of displaying real-world health care services, and little is known about how such eHealth tools can improve access to care.

**Objective:**

This paper describes the processes of co-creating and evaluating the beta version of a Web-app aimed at mapping and describing free or low-cost real-world health care services available in the Bordeaux area of France, which is specifically targeted to university students.

**Methods:**

The co-creation process involves: (1) exploring the needs of students to know and access real-world health care services; (2) identifying the real-world health care services of interest for students; and (3) deciding on a user interface, and developing the beta version of the Web-app. Finally, the evaluation process involves: (1) testing the beta version of the Web-app with the target audience (university students aged 18-30 years); (2) collecting their feedback via a satisfaction survey; and (3) planning a long-term evaluation.

**Results:**

The co-creation process of the beta version of the Web-app was completed in August 2016 and is described in this paper. The evaluation process started on September 7, 2016. The project was completed in December 2016 and implementation of the Web-app is ongoing.

**Conclusions:**

Web-apps are an innovative way to increase the health literacy of young people in terms of delivery of and access to health care. The creation of Web-apps benefits from the involvement of stakeholders (eg, students and health care providers) to correctly identify the real-world health care services to be displayed.

## Introduction

### Overview

The years spent in university are a time of increasing independence and growth for young people. During this period, students actively make decisions about their health care and the healthy (or unhealthy) behaviors that they wish to adopt [1]. To prevent the risk of several diseases, young people aged 18-30 years are encouraged to regularly consult a general practitioner (and a gynecologist for females) in addition to consultants for particular health conditions (eg, dentists or ophthalmologists) [2]. Notwithstanding national recommendations and health promotion programs, French university students underuse health care services, with 20% not consulting a health professional (general practitioner or specialist) during their university years [3]. A small number of international studies have examined why young people avoid and delay medical care [4], providing a conceptual categorization of three main barriers: low perceived need to seek medical care; traditional barriers to medical care such as high cost, absence of health insurance, and time constraints; and lack of knowledge concerning the organization of the health care system and its services [5]. A study conducted on 41,000 French university students reported that: 23% of the participants did not feel the need to seek medical care, 13% did not have time for medical consultations, and 12% had economic difficulties to access and pay for health care services [3]. Another study examining 2000 French young adults (not all students) aged 15-30 years reported that lack of knowledge concerning the organization of the health care system and its services is a significant factor hindering utilization of health care services for 30% of young participants [6]. Our study took these three barriers into account with a specific focus on lack of knowledge as one component of students’ *health literacy*, namely the lack of acquired and assimilated information on how to access health care [7]. Usually supported and guided by their parents in the management of their health consultations, young people moving away from home to start their university studies face, for the first time, the need to find a health care service, contact it, and access it on their own.

In parallel, university students are a technologically capable generation, having been born and raised in the age of home computers and portable electronic devices [8]. Using new technologies for obtaining information on the availability of health care services could represent an appealing solution for increasing students’ health literacy.

However, notwithstanding their high use of new technological devices [9], young people have expressed their concerns about the quality and utility of existing electronic health (eHealth) tools [10]. Similarly, professionals coming from real-world health care services sometimes perceive eHealth solutions as complicating the health care provider-patient relationship [11], and being an unreliable source for medical advice [12].

A small number of eHealth devices have been evaluated to date [13]. Most of these devices have been produced by Web-developers that have little experience of health care, and have not taken stakeholders’ opinions into consideration. More specifically, eHealth devices proposed as a bridge between eHealth and real-world health care are still scarce. Very little is known about the possible association between the use of eHealth tools and the use of real-world health care services, especially among young people [14].

We embarked on co-creating and evaluating a Web-app available on laptops, personal computers, smartphones, and tablets to show university students of the Bordeaux area of France low-cost or free health care services at their disposal, and where these services are exactly located. This is the local Bordeaux example of Web-app that could be extended at the national level in other French universities. The iterative processes of co-creating and evaluating the Web-app called the *services for the Internet-Based Students Health Research Enterprise (i-Share) students’ cohort* (servi-Share) are described in this paper.

### The servi-Share Project

The servi-Share project is nested in the larger i-Share cohort study, which is a nationwide online survey on the health and well-being of French-speaking university students. The i-Share cohort study started in 2013 from the collaboration of the University of Bordeaux and the University of Versailles Saint-Quentin (France), and is still ongoing across France. To be eligible to participate, students must be officially registered at a university or higher education institute, be at least 18 years of age, be able to read and understand French, and provide online consent for participation. The i-Share study was approved by the Commission Nationale de l'Informatique et des Libertés (CNIL; National Commission of Informatics and Liberties; DR-2013-019).

Preliminary analyses were conducted in the third year of the cohort study, on a total of 8770 students, 6578 of whom were females (75.01%). Results showed relatively high percentages of avoidance and delay of medical care: 3251 students (37.07%) declared having gone without recommended care, notwithstanding the need to see a doctor (general physician, consultant, eye-specialist); 1316 students (15.01%) declared having not seen a dentist, notwithstanding the need for a consultation; and 1325 students (15.11%) reported having gone without complementary health exams (ie, blood sample, radiography) prescribed by a doctor. Given these high rates, we explored the opportunity to put into practice a Web-based intervention aimed at facilitating students’ access to real-world health care services. The servi-Share project was then implemented. First, a beta version of the servi-Share Web-app was developed and tested by students. Second, considering the results of the beta version tests, the Web-app will be corrected and implemented to be openly and largely diffused to university students within the Bordeaux area.

The two main hypotheses underlying the servi-Share project are that: (1) co-creating and evaluating a health Web-app with stakeholders may contribute to the production of an effective quality eHealth device, and (2) a better-quality eHealth device mapping real-world health care services should increase young people’s health literacy in terms of knowledge of and access to health care.

## Methods

The production of the Web-app consisted of two main processes: (1) co-creation, and (2) evaluation. Each process consisted of further operational stages involving academic staff and industry Web-developers, together with two target stakeholder groups (university students and real-world health care service providers). The two processes used both qualitative and quantitative methods. We opted for the co-creation process to involve stakeholders from the very beginning of the project, and not as mere testers of the finished Web-app [15]. The goal of this approach was to produce a Web-app that met the real needs of students, and corresponded to the precise choices of real-world health care service providers.

### Process 1: The Co-creation

#### Exploring the Need of Students to Know and Access Real-World Health Care Services

A mixed-method field survey was conducted on the campuses of the University of Bordeaux. Participants were selected randomly following a quota sampling for the quantitative phase (paper questionnaire), and a snow-balling approach for the qualitative phase (semistructured face-to-face interviews). Our sampling strategy and rationale for the number of participants were based on previous project experience with university students, who declared that they were often unavailable, given their workload. At least 100 respondents to the questionnaire and 15 participants in the qualitative phase were considered sufficient to obtain a saturated sample. Finally, 126 students (72 females, 57.1%; mean age 22.1 years) answered the paper questionnaire and 16 students (11 females, 69%; mean age 22.3 years) underwent the semistructured face-to-face interview. The survey was coordinated by a junior full-time researcher and conducted by a group of four public health students (1 male and 3 females; mean age 23.7 years), constituting the stakeholder group of university students for the project. The results of this survey showed that students had the feeling that accessing real-world health care services is an expensive practice (59/126, 46.8%) which takes time (49/126, 38.9%). The qualitative phase allowed for the identification of a third overarching reason for students not to access to care: lack of knowledge of the health insurance system and the services offered. Two thirds of the students from both phases of the survey reported a strong interest in receiving a list of free or low-cost health care services available near their home and campus. The French health insurance system reimburses a large portion of medical consultations [16], but students expressed the need to be informed of the presence of totally free health care services adapted to their young age. Furthermore, receiving this list from a trusted source, such as a university research team, was felt to be reassuring. Complete results of the mixed-method field survey are available elsewhere (personal communication by Montagni et al, 2016).

#### Identifying the Real-World Health Care Services of Interest for Students

Based on the results of the survey described above, we established the following inclusion criteria for the real-world health care services to be displayed in the Web-app: being located in the Bordeaux metropolitan area (surface area 579,27 km^2^; 28 municipalities in the Aquitaine-Limousin- Poitou-Charentes region, France); being free or low-cost (ie, costing a maximum of €15 per consultation); being addressed, either exclusively or among other population groups, to young people aged 18-30 years; and being outpatient. All health domains were taken into consideration without any exception (from general health to sexual health, gynecology, and dentistry). Emergency services were excluded, because the focus of the Web-app related to recommended general consultations.

At this stage, both target stakeholder groups selected by the servi-Share project were involved. For university students, the four public health students of the first stage performed a qualitative search consisting of a preliminary revision of existing documents (fliers, informative booklets), and the consultation of official Websites of the University of Bordeaux and local health services. These students produced an initial list of services in a prestructured Excel table and contacted each one by email and/or phone service. This phase served to produce a final list of 95 services distinguished according to their field of expertise (eg, addictions, contraception), their offer (eg, consultations, delivery of information, medical activities), and the type of professionals (eg, medical doctors, nurses, social carers). For each service, contact information and addresses were provided.

The target stakeholder group of real-world health care service providers was composed of seven health care professionals based in Bordeaux (1 health center director, 1 health center codirector, 1 psychiatrist, 1 general practitioner, 1 social worker, 1 administrative secretary, and 1 nurse). The stakeholder group of real-world health care service providers verified the list and counter-checked the details with respect to the inclusion criteria. After face-to-face meetings between the two target stakeholder groups, a final list of 88 health care services was established. Assuming that the Web-app will be maintained on the long term, we plan to contact the panel of seven health care professionals based in Bordeaux once per year to review and keep the list of health care services up-to-date. These professionals are also meant to inform our research group anytime new health care services are created or old health care services are closed.

#### Deciding on User Interface, and Developing the Beta Version of the Web-App

Academic staff and industry Web-developers involved in the project engaged the stakeholder group of four university students in the development of the Web-app. The four students participated in three 2-hour meetings with the industry Web-developers. Sessions were documented and students were encouraged to write on material provided, comment on the color templates, and suggest design decorations. Subsequent exchanges were facilitated by emails and phone calls.

**Figure 1:**
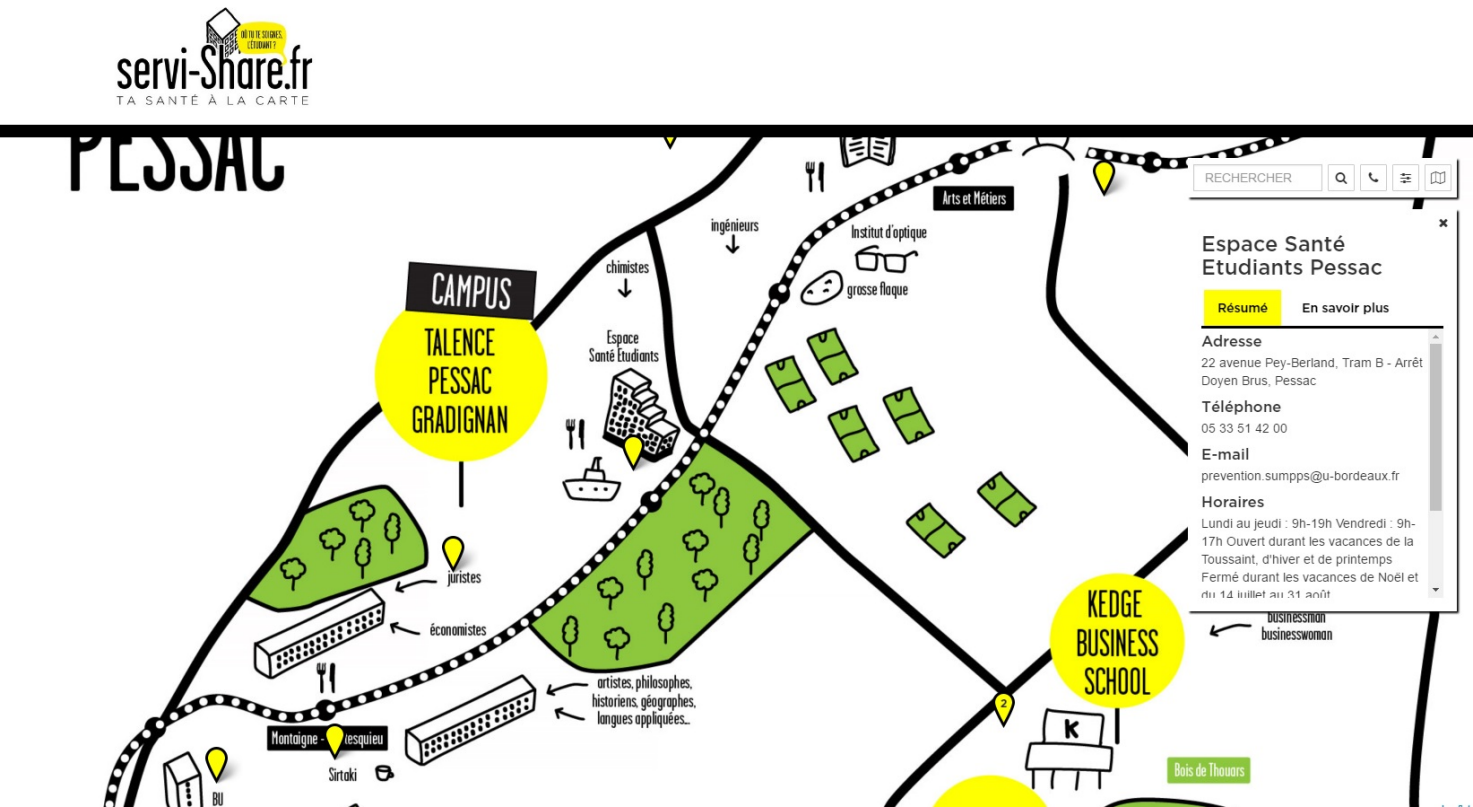
Example of description of a service displayed in the servi-Share Web-app.

For the stakeholder group of real-world health care service providers, their intervention at this stage was limited to the verification of information to be displayed in the Web-app. The stakeholder group of real-world health care service providers checked the information for clarity and comprehension, and corrected the descriptions of the real-world health care services. The correct contents were sent by email to the Web-developers. Figure 1 shows how description of the services is provided in the beta version of the servi-Share Web-app.

**Figure 2:**
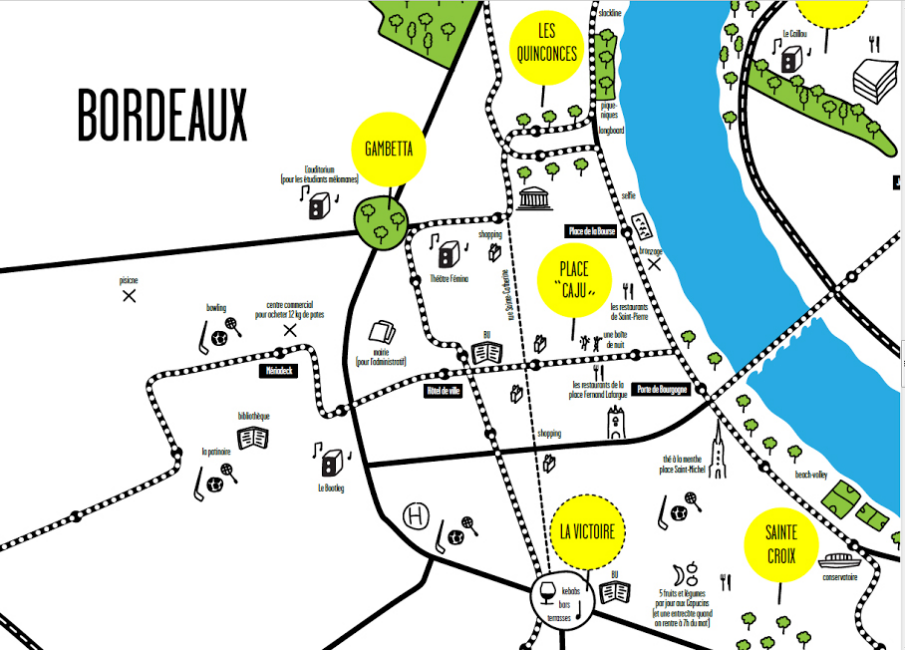
Detail of the map layout in the servi-Share Web-app.

We finally opted for a youth-friendly approach [17], balancing trusted informative content and a fresh design. Feedback at this stage was specifically sought regarding the colors, size, readability, and comprehension of the text and design elements (eg, logo, symbols). Students primarily had suggestions regarding specific design constructs, recommending graphic design to be gender neutral, with positive images and a simple interface. The beta version of the servi-Share Web-app was then produced. A detailed view of the beta design is shown in Figure 2.

### Process 2: The Evaluation

#### Testing the Beta Version of the Web-App with Target Audience

An initial one-week test was performed by research staff from the i-Share study: 7 people tested the Web-app to report technical problems (eg, slow page loading), misspellings, and display issues. The second test of the beta version of the Web-app took two months and consisted of emailing the Web-app link to a sample of students belonging to the i-Share cohort study, in which the servi-Share project is nested. Students participating in the i-Share cohort study had voluntarily given their email addresses, and consented to be contacted following the regulations of the national board CNIL (DR-2013-019). We chose a convenience sample of 1300 students based on the following criteria: being a student in one of the universities or post-secondary schools of the Bordeaux area, being aged 18-30 years, and having completed both the i-Share baseline questionnaire and first follow-up questionnaire. Students responding to the follow-up questionnaire were thought to be actively involved in research, thus increasing the possibility of having their rapid feedback. According to the general response rate of i-Share participants to other substudy surveys (approximately 20%), we expected at least 300 students to use the Web-app and answer a 7-item questionnaire on the access to real-world health care services (see Multimedia Appendix 1). This sample did not include students from the co-creation process, to avoid bias in the answers and have new feedback on the Web-app.

The beta test was intended to develop a better understanding of how the Web-app was being used, and what issues arose during implementation. Student testers were asked to independently interact with the interface and search for real-world health care services on the Web-app. For ethical reasons, participants’ usage of the Web-app could not be registered and health care services that were searched could not be tracked. The beta version of the Web-app is secured and password protected for students having received the link in the invitation email. Of the 1300 invited students, a final sample of 319 students tested the Web-app (24.54%).

#### Collecting Testers’ Feedback Via a Satisfaction Survey

At the end of the test of the beta version (early November 2016), a satisfaction survey was sent by email to all 319 student testers. The survey was carried out using a Google form and included 10 items on six thematic issues: feasibility (item 1, “Have you encountered any difficulties in using this Web-app?”); appreciation of the Web-app (item 2, “Do you like the Web-app design?”, and item 10, “How would you rate this Web-app?”); increased knowledge and perceived benefits (item 3, “Have you discovered through this Web-app some health care services you had never heard about before?”, and item 4, “Have you found in the Web-app some new health care services you will have access to for the future?”); general interest (item 5, “Will you use this Web-app in the future instead of other geolocalization search engines if you need to contact a health care service?”, and item 8, “Will you recommend this Web-app to your friends?”); diffusion channels (item 6, “Where would you like to see this Web-app being promoted and diffused?”, and item 7, “Through which channels do you think that students should be informed of the existence of this Web-app?”), and suggestions for improvement (item 9, “What would you like to add to this Web-app?”).

The satisfaction survey represented the first step of the evaluation of the servi-Share Web-app. Using a participatory research methodology consisting of an iterative approach, we involved stakeholders in the short-term evaluation of the Web-app. In total, 73 of 319 students (22.9%, no missing values) answered the satisfaction questionnaire. Results are shown in Table 1.

**Table 1:** Results of the satisfaction questionnaire (n=126).

Thematic issue	Items	Yes	No
1. Feasibility	Item 1 - Have you encountered any difficulties in using this Web-app?	6 (8%)	67 (92%)
2. Appreciation	Item 2 - Do you like the Web-app design?	55 (75%)	18 (25%)
3. Increased knowledge and perceived benefits			
	Item 3 - Have you discovered through this Web-app some health care services you had never heard about before?	62 (85%)	11 (15%)
	Item 4 - Have you found in the Web-app some new health care services you will have access to for the future?	61 (84%)	12 (16%)
4. General interest			
	Item 5 - Will you use this Web-app in the future instead of other geolocalization search engines if you need to contact a health care service?	50 (68%)	23 (32%)
	Item 8 - Will you recommend this Web-app to your friends?	51 (70%)	22 (30%)

Concerning the diffusion channels (items 6 and 7), 54 of 73 students (74%) answered that they would like the Web-app to be displayed on the official website and social network pages of their university, 40 students (40/73, 55%) would not mind finding the Web-app on GooglePlay and/or AppStore, and 70 students (70/73, 96%) underlined the importance of diffusing the Web-app via the support of official institutions (eg, university and town hall).

Concerning suggestions for improvement (item 9), 65 of 73 students (89%) said the Web-app should display supplementary health care services, such as general practitioners and pharmacies, and 55 students (55/73, 75%) also reported that they would like to make an appointment online using the Web-app. Finally, when asked to rate the Web-app (item 10) on a scale from 0 to 10 points, 61 students (61/73, 84%) attributed a score of >7 points.

Results confirmed the interest of developing and diffusing a Web-based support informing students on the availability of free or low-cost health care services. Particularly positive results on items 3 and 4 confirmed students’ acquired knowledge (health literacy) of the real-world health care services in the Bordeaux area, providing a proximal outcome of the utilization of our Web-app.

#### Planning a Long-Term Evaluation

For the second step of the evaluation, the real impact of the Web-app on users’ health behaviors and practices in the long-term will be measured via the analysis of the i-Share cohort data. Each year, participants in the i-Share cohort must respond to a new yearly follow-up questionnaire. We plan to insert ad hoc items on at least two upcoming questionnaires to verify whether students have ever used the servi-Share Web-app and what impact it has had on their consultations and hospitalizations. Statistics on the number of participants accessing the Web-app will also be available and will give an approximative indication of the popularity of the Web-app. To corroborate our results, in the two years following the launch of the Web-app, the providers of the real-world health care services displayed in the Web-app will be asked (by means of a questionnaire) whether young people accessing their services have used our interactive map before consultations. All measures coming directly from stakeholders should provide a complete evaluation of the utility and quality of the servi-Share Web-app.

## Results

The co-creation process took a total of 8 months (January-August 2016). The first step of the evaluation process has taken 4 months (September-December 2016). The second step of the evaluation (ie, long-term impact) is planned to take two years. Results are expected to contribute to the evidence-based development of a strategy of cooperation and collaboration among researchers, stakeholders (students and health care providers), and industry to produce eHealth tools of good and certified quality. The project was financed from January-December 2016 by the National Alliance for Life and Health Sciences (Alliance Nationale pour les Sciences de la Vie et de la Santé, AVIESAN) through two research financing Thematic Multi-Organisms Institutes (Instituts thématiques multi-organismes, ITMO) for Public Health (ITMO Santé Publique) and Health Technologies (ITMO Technologies pour la Santé).

## Discussion

Here we have outlined the co-creation and evaluation processes used during the development of a Web-app mapping real-world health care services. The methodologies of the singular stages of these two processes have also been described, and we have underlined the utility of including stakeholders in both processes. The philosophy underpinning the servi-Share project is one of collaboration, empowerment, and participation, moving towards research *with* rather than *on* stakeholders.

For co-creation, the participatory approach with stakeholders was effective for informing design and development processes to help ensure our project is relevant, connects with young people, is grounded in the real-world, and can respond to the new social realities in which students live [18].

For evaluation, the two steps of this process permit us to assess: (1) in the short-term, if the Web-app is of interest to students and increases their health literacy in terms of knowledge of real-world health care services (proximal outcomes); and (2) in the long-term, if the Web-app will imply behavioral changes that make students more frequently contact and access real-world health care services (distal outcomes). The second step of the evaluation process will allow us to test whether the servi-Share Web-app represents a valid bridge between eHealth and real-world services, and whether other functions (eg, making appointments online) should be added to the Web-app.

Existing literature on health literacy strongly suggests that young people’s health empowerment is induced by knowledge improvement [19,20]. However, the transition from knowledge to action is a debatable question. The longitudinal results issued from the i-Share cohort will help us understand whether the use of the geolocalizing Web-app servi-Share is positively related to the access to real-world health care services (ie, an increased number of accesses to real-world health care services). Using our Web-app, we hypothesize that students will better understand the organizational structure and offerings of real-world health care services, thus identifying the health care services to promptly contact and attend. Avoidance and delay of medical care could then be reduced.

### Strengths and Limitations

The servi-Share Web-app is different from other Web-mapping tools (eg, Google Map, Waze Map, Bing Map) that display any type of service without specific quality criteria, as it maps and describes preselected real-world health care services. This preselection should facilitate the choice by users, who can feel reassured by the fact that the health care services that are displayed are specifically addressed to them, are free or low-cost, and are advised by an expert scientific team.

However, a limitation of this study is that one may argue that a Web-app showing real-world health care services could increase the workload of these services, and overwhelm them with contacts that do not correspond to legitimate health needs. The aim of the servi-Share Web-app is not to increase undue consultations, but to guide students to a well-conceived selection of the services to access. Conversely, the servi-Share Web-app has not been conceived as a substitute for real-world health care services. The young population we are addressing is at risk for medical care avoidance. Most students do not know who to contact when they fall ill, and consequently do not seek care and tend to self-medicate, thus worsening their health conditions [21].

### Conclusions

We have described a novel approach using a Web-app linking real-world health care services and eHealth. Our preliminary findings concerning the co-creation process suggest that this participatory approach was both feasible and welcomed by both groups of stakeholders (university students and real-world health care service providers). Findings from the evaluation process will assess the long-term impact of the Web-app on real-world health care access by university students. Our Web-app is expected to be beneficial to young people, health care providers, policymakers, and health system managers.

## Appendix

Multimedia Appendix 1 The web-application 7 item questionnaire on the access to real-world healthcare services .

## Abbreviations

CNIL: National Commission of Informatics and Liberties
eHealth: electronic health
i-Share: Internet-Based Students Health Research Enterprise
ITMO: Thematic Multi-Organisms Institute
servi-Share: services for the Internet-Based Students Health Research Enterprise students’ cohort
