# Supplementary material for: Co-creating and Evaluating a Web-app Mapping Real-World Health Care Services for Students: The servi-Share Protocol
Source: JMIR Res Protoc. 2017 Feb 16;6(2):e24. doi: 10.2196/resprot.6801 (PMC5334513; doi:10.2196/resprot.6801)

Appendix 1

The web-application 7 item questionnaire on the access to real-world healthcare services

Question 1: Where do you live?

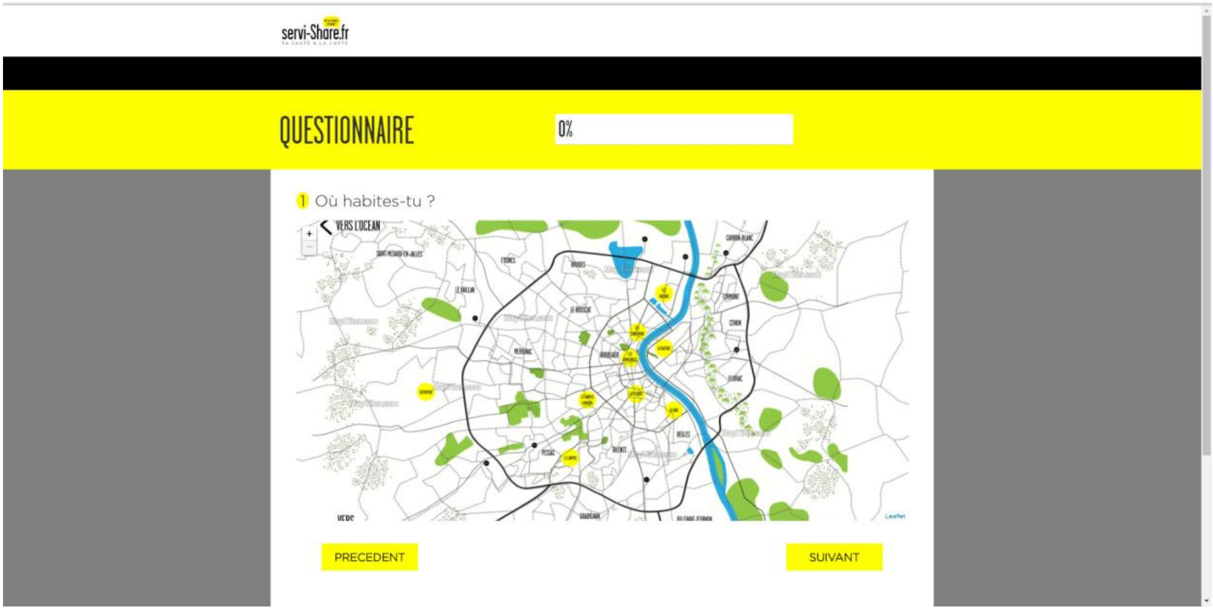

Question 2: Where do you study?

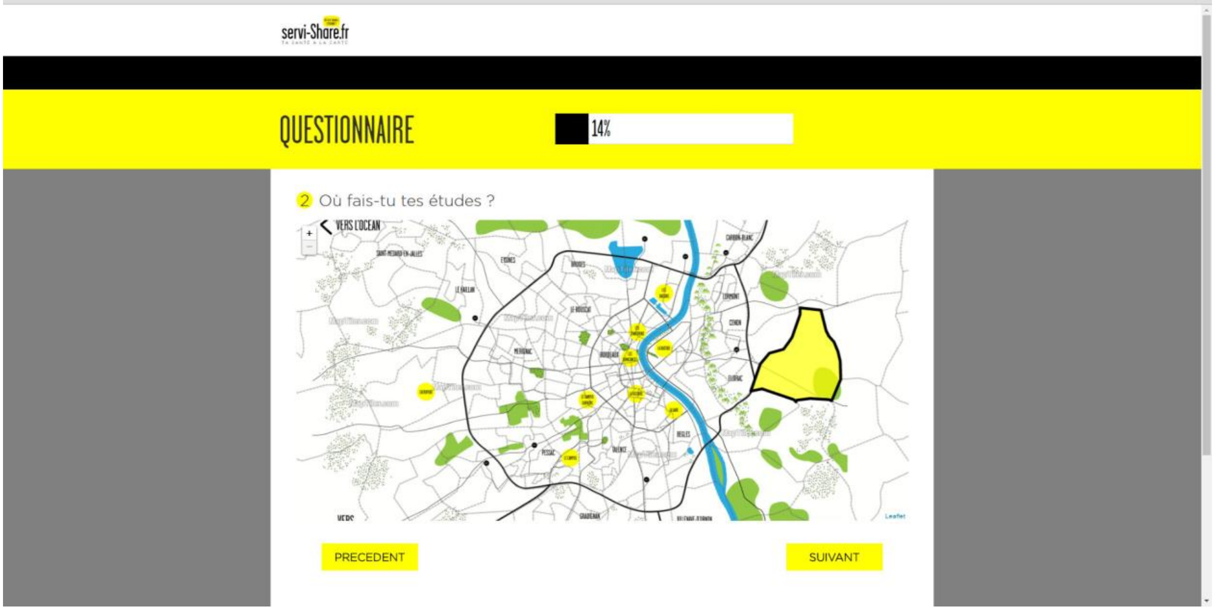

Question 3: How long have you been living in the Bordeaux area?

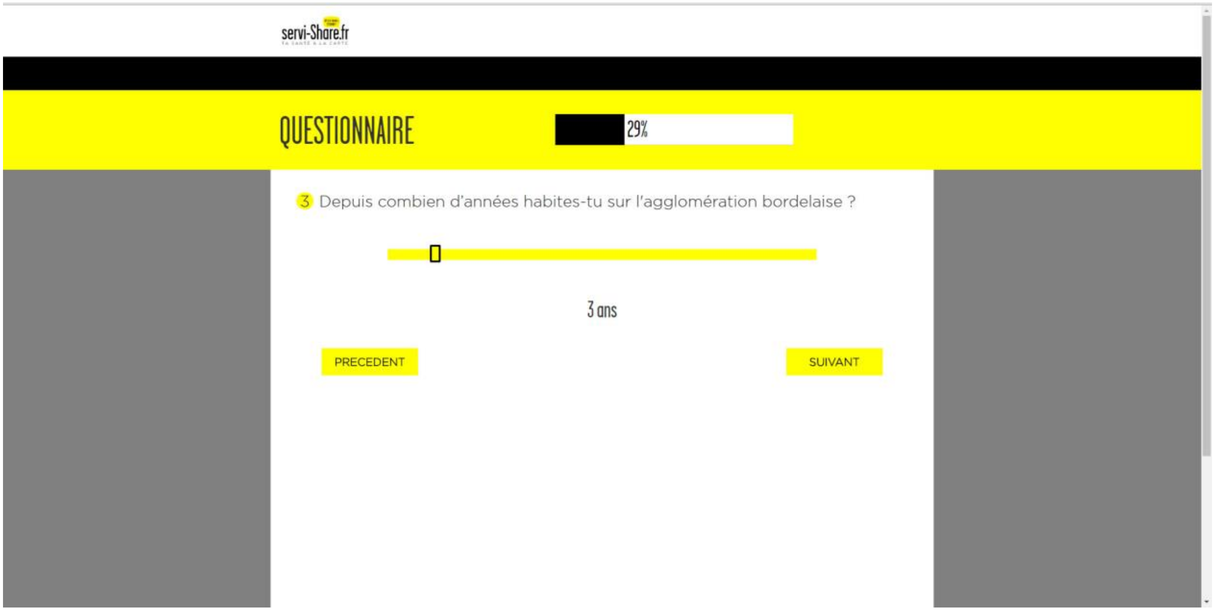

Question 4: Which are your means of transport?

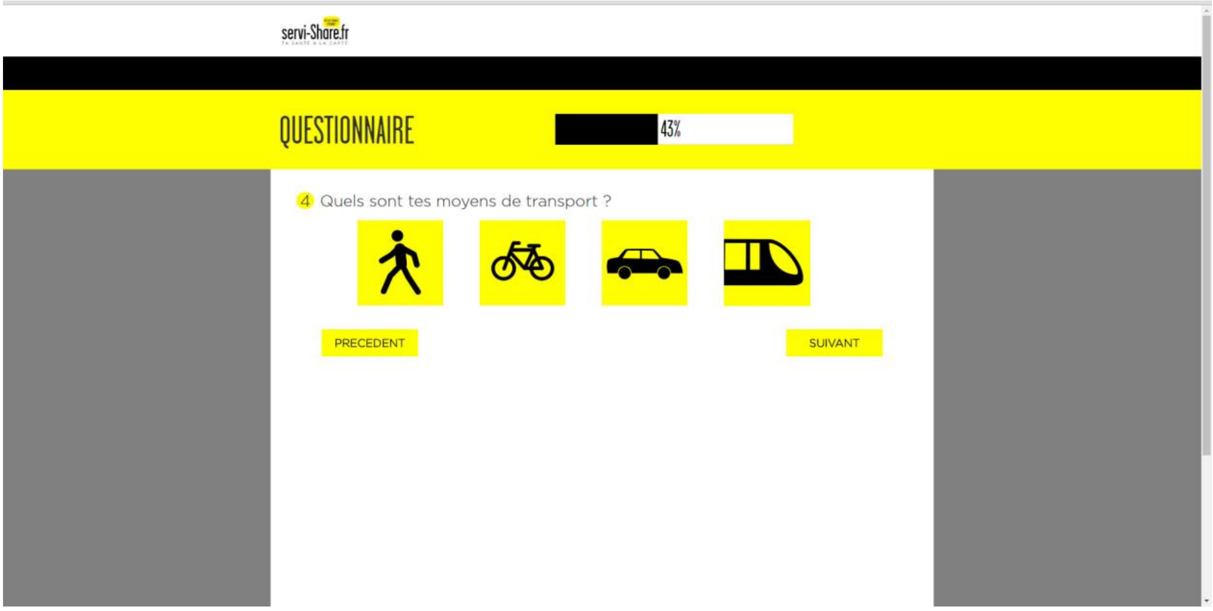

Question 5: Which type of healthcare services are you looking for?

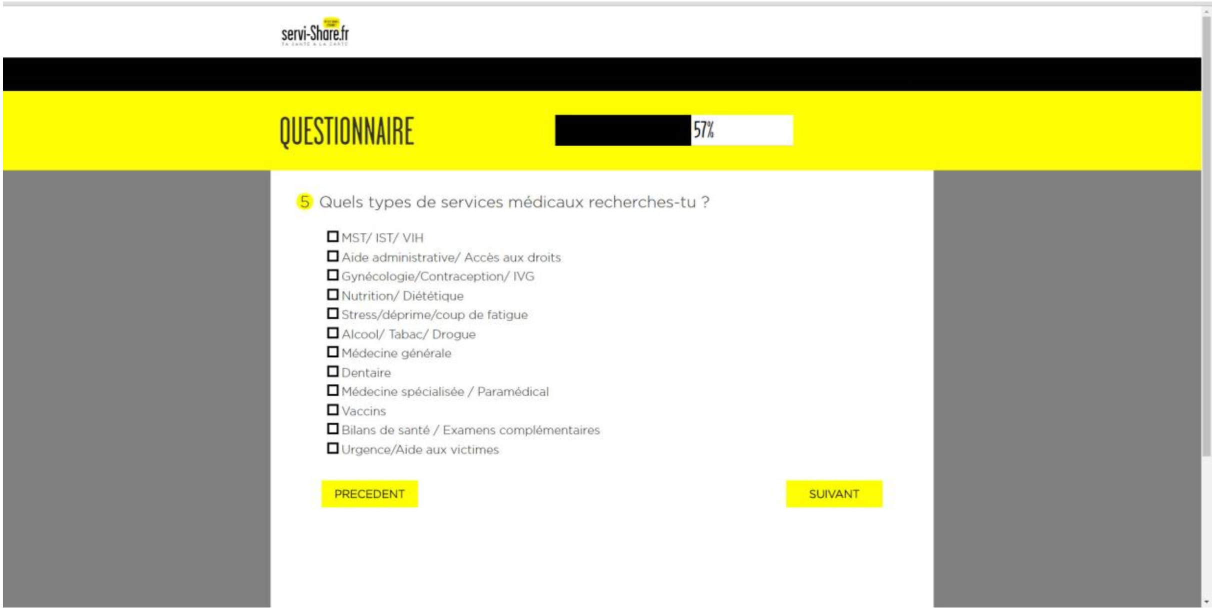

Question 6: Which are your criteria to choose a healthcare service?

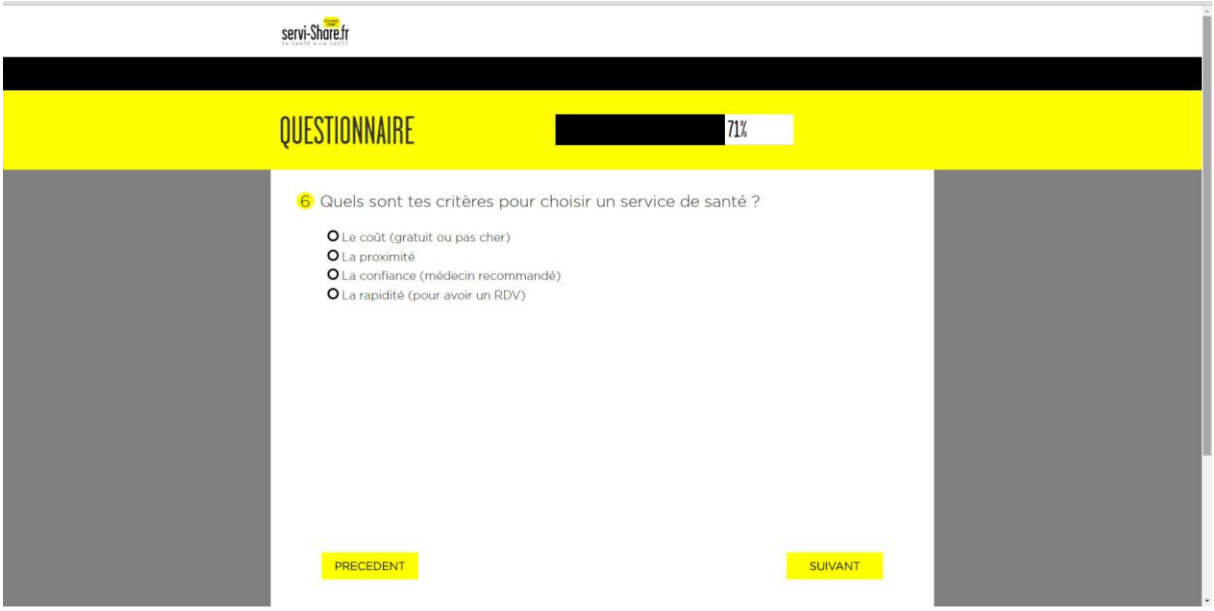

Question 7: Do you know any fee-for-free healthcare service in the Bordeaux area?

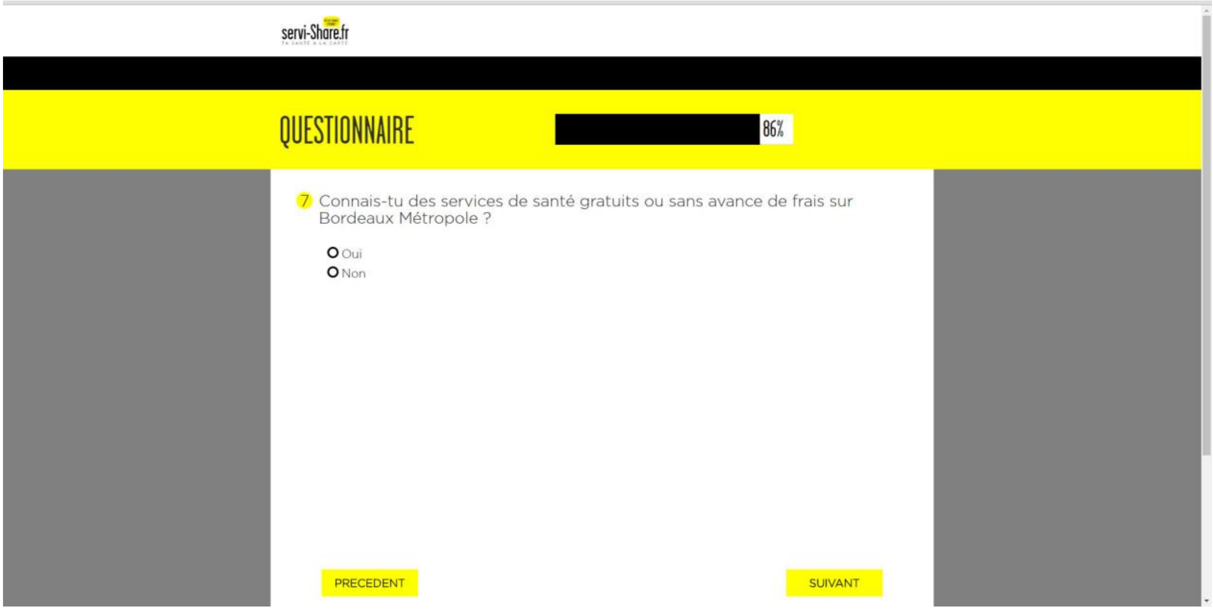

Supplement: Multimedia Appendix 1 [file resprot_v6i2e24_app1.pdf]
